# Metabolic Syndrome Is Associated With Poor Cognition: A Population-Based Study of 70-Year-Old Adults Without Dementia

**DOI:** 10.1093/gerona/glab195

**Published:** 2021-07-06

**Authors:** Anna Marseglia, Alexander Darin-Mattsson, Johan Skoog, Lina Rydén, Timothy Hadarsson-Bodin, Silke Kern, Therese Rydberg Sterner, Ying Shang, Anna Zettergren, Eric Westman, Ingmar Skoog

**Affiliations:** 1 Division of Clinical Geriatrics, Center for Alzheimer Research, Department of Neurobiology, Care Sciences and Society, Karolinska Institutet, Stockholm, Sweden; 2 Aging Research Center, Department of Neurobiology, Care Sciences and Society, Karolinska Institutet and Stockholm University, Sweden; 3 Centre for Ageing and Health (AgeCap), Neuropsychiatric Epidemiology Unit, Department of Psychiatry and Neurochemistry, Institute of Neuroscience and Physiology, Sahlgrenska Academy at the University of Gothenburg, Mölndal, Sweden; 4 Department of Psychology, University of Gothenburg, Gothenburg, Sweden; 5 Department of Psychiatry Cognition and Old Age Psychiatry, Sahlgrenska University Hospital, Region Västra Götaland, Mölndal, Sweden

**Keywords:** Apolipoprotein E4, Cardiovascular disease, Education, Vascular cognitive impairment

## Abstract

**Background:**

Individual conditions of metabolic syndrome (MetS) have been related to dementia; however, their combined impact on the preclinical stage is unknown. We investigated the associations between MetS and domain-specific cognitive function as well as the role of sociodemographic, cardiovascular, and genetic factors.

**Methods:**

Within the Gothenburg H70 Birth Cohort Study-Birth cohort 1944, 1131 dementia-free participants (aged 70 years) were examined during 2014–2016. MetS (central obesity plus at least 2 factors [reduced HDL-cholesterol, elevated triglycerides, blood pressure, or blood glucose]) was identified according to the International Diabetes Federation criteria. Five cognitive domains (memory, attention/perceptual speed, executive function, verbal fluency, visuospatial abilities) were generated after *z*-standardizing raw scores from 10 neuropsychological tests. Education, heart disease, claudication (indicating peripheral atherosclerosis), and apolipoprotein genotype were ascertained by trained staff. Data were analyzed with linear regression models.

**Results:**

Overall, 618 participants (55%) had MetS. In multiadjusted linear regressions, MetS was related to poorer performance in attention/perceptual speed (β −0.14 [95% CI −0.25, −0.02]), executive function (β −0.12 [95% CI −0.23, −0.01]), and verbal fluency (β −0.19 [95% CI −0.30, −0.08]). These associations were present only among individuals who did not carry any *APOE*-ε4 allele or were highly educated. However, among those with MetS, high education was related to better cognitive performance. MetS together with comorbid heart disease or claudication was associated with even worse cognitive performance than each alone.

**Conclusions:**

MetS is associated with poor attention/perceptual speed, executive function, and verbal fluency performance. Education, apolipoprotein E-ε4 allele, and comorbid cardiovascular disease influenced the observed associations.

Dementia is a common disorder in the aging population and the number of people with dementia is expected to increase in the next 30 years (1). Metabolic disorders (eg, obesity and diabetes) are among the major risk factors for dementia and do not typically occur alone, but in aggregation with other cardiovascular risk factors, such as hypertension and dyslipidemia, defining the metabolic syndrome (MetS) (2,3). The prevalence of MetS parallels that of obesity and diabetes—up to 45% of the population worldwide—and is also expected to rise as a consequence of increasing longevity and unhealthy lifestyles (2,3). Because of the lack of a pharmacological cure for dementia, addressing 2 of the most burdensome age-related diseases (MetS and dementia), together, is important to identify ideal targets and strategies for dementia prevention.

Each component of MetS (eg, obesity, diabetes, and hypertension), either in midlife or later in life, has been consistently related to increased dementia risk (4–6). However, the combined contribution of these conditions in the early stages of deteriorating cognitive health remains unclear. MetS has been related to dysfunction in global cognition and executive function in some previous studies (7–9), but not in other studies (10,11). Yet, a possible link between MetS and other cognitive domains (eg, memory, language, visuospatial abilities) remains controversial (7,12).

Cardiovascular and cerebrovascular diseases (ie, heart disease and stroke) are common comorbidities of metabolic conditions such as diabetes and obesity. For example, it has been estimated that about 35% of people with diabetes are affected also by cardiovascular and cerebrovascular diseases (13). Besides, cardiovascular disease promotes pathophysiological alterations in the large and small vessels, which can lead to cognitive impairment and dementia over time (14). However, whether the co-occurrence of MetS and cardiovascular diseases may have amplified consequences on cognition has not been explored before. A potential mechanism underlying cognitive dysfunction that is shared by both MetS and cardiovascular disease is atherosclerosis (15,16). Claudication is a symptom of clinically manifested peripheral artery disease, in which the arteries that supply blood to the limbs are progressively narrowed because of atherosclerosis (17). Therefore, claudication can be used as a marker to address the role of atherosclerosis in MetS-associated cognitive dysfunction.

In this study, we aimed to investigate (i) the associations between MetS and domain-specific cognitive function and (ii) whether education, cardiovascular disease, and the apolipoprotein E-ε4 (*APOE*-ε4) allele, a genetic risk factor for Alzheimer’s disease (AD), could modify such associations, among a population-based cohort of 70-year-old people without dementia.

## Methods

### Study Design, Setting, and Participants

This cross-sectional study included participants from the baseline assessment of the ongoing multidisciplinary study on health and aging, the Gothenburg H70 Birth Cohort Studies (H70)–Birth cohort 1944 (18). The study design and procedure of the H70–Birth cohort 1944 has been reported in detail elsewhere (18). Briefly, all individuals born in 1944 (aged 70 years) and residents of Gothenburg, Sweden, were identified through the Swedish Tax Agency’s population register and invited to participate in the baseline examination (January 2014–December 2016), regardless of place of living (eg, home, institutions). Of the 1839 initially eligible for participation, 29 died before examination and 607 declined to participate, leaving 1667 people who were invited to participate in the baseline examination. Of the 1203 who agreed to participate in the study (response rate 72.2%), we excluded individuals with dementia, Mini-Mental State Examination (MMSE) score < 24, Parkinson’s diseases, normal pressure hydrocephalus, or multiple sclerosis (*n* = 64), and with missing information on the MetS (*n* = 8), leaving 1131 participants for the current study (see Supplementary Figure 1).

The Regional Ethical Review Board in Gothenburg approved all parts of the H70 study. All participants or their next-of-kin (if the participant was unable to provide own consent) provided written informed consent.

### Data Collection

Information on sociodemographics (sex and education), cardiovascular risk factors (blood pressure, smoking, alcohol consumption, and physical activity), anthropometrics (eg, height and weight), somatic and psychiatric conditions and symptoms, and current medication use were collected through semistructured interviews and clinical examinations by research nurses or medical doctors.

Education was categorized into primary school (primary and lower secondary education with <9 years of schooling), secondary school (9 years of schooling or ≤2 years of vocational training), or higher education (>2 years of vocational training or university). Smoking was dichotomized into never smoked versus current/former smoking. At-risk alcohol consumption was identified as ≥100-g alcohol/wk, which corresponded to the National Institute on Alcohol Abuse and Alcoholism’s definition of heavy consumption for women and men aged 65 and older (19). Body mass index was categorized into underweight (<20 kg/m^2^), normal weight (20 to <25 kg/m^2^), overweight (25 to <30 kg/m^2^), or obese (≥30 kg/m^2^). Systolic and diastolic blood pressure was measured in the right arm in the sitting position after 5-minute rest using a manual sphygmomanometer (18).

Physical activity was dichotomized into inactive (ie, no physical activity or sedentary most of the day/nonregular lighter walks) versus active (ie, regular nondemanding physically activities [eg, walks, gardening, dancing] 2–4 times per week, demanding physical activities [eg, tennis, running, swimming] at least 1 h/wk, or hard regular exercise). Proxy-informant interviews were conducted by a psychologist or research nurse and included questions on symptoms and signs of dementia and stroke/transient ischemic attack (TIA). Diagnoses of medical conditions were based on multiple sources—which were examinations, self-report, medication use, or information from the Swedish National Patient Register (NPR). The NPR is coded according to the International Classification of Diseases—10th edition (ICD-10) and includes hospital discharge diagnoses and specialized outpatient care. Medical conditions included cardio- and cerebrovascular diseases—that is heart diseases (myocardial infarction, angina pectoris, heart failure, atrial fibrillation, and stroke/TIA)—and intermittent claudication (for details on the assessments, see Supplementary Appendix A) (20). Depression (minor and major) was diagnosed according to the *Diagnostic and Statistical Manual of Mental Disorders* (DSM)—4th or 5th editions criteria as previously described (21). Dementia was diagnosed according to the DSM—3rd revised edition using information from the neuropsychiatric examination and the key informant interview at a consensus meeting by psychiatrists, as described previously (22,23). In the current study, dementia was only used as exclusion criteria. Blood samples were taken from all participants, either after fasting (at least 8 hours of no caloric intake, *n* = 1096) or nonfasting status (*n* = 30) (24). Diabetes was identified based on self-reported medical history, use of glucose-lowering treatments (diet, oral hypoglycemic agents, or insulin), or fasting/nonfasting blood glucose of ≥7.0/11.1 mmol/L (25). DNA was extracted from blood samples. *APOE* single-nucleotide polymorphism was genotyped, and participants divided into ε4 carriers versus noncarriers.

### Assessment of MetS

MetS was identified according to the International Diabetes Federation criteria (2) including the presence of central adiposity (waist circumference of ≥94 cm in men or ≥80 cm in women), plus at least 2 of the following 4 factors: (i) raised triglycerides (≥1.7 mmol/L or use of lipid-lowering medication [Anatomical Therapeutic Chemical Classification System code C10]); (ii) reduced high-density lipoprotein cholesterol (<1.03 mmol/L in men and <1.29 mmol/L in women); (iii) raised blood pressure (systolic/diastolic blood pressure ≥ 130/85 mm Hg or current antihypertensive treatment); and (iv) raised blood glucose (fasting/nonfasting plasma glucose ≥5.6/≥7.8 mmol/L or diagnosed diabetes).

### Assessment of Cognitive Domains

Cognitive assessments were performed by research nurse, trained research staff, psychiatrist, or medical doctor under the supervision of a neuropsychologist. A neuropsychological test battery, comprised of 10 tests, wan administered to all participants (18). The battery addressed 5 core cognitive domains: *episodic memory* (derived from the Memory in Reality–free recall and 12-object delayed recall, and Thurstone’s picture memory) (26,27), *attention/perceptual speed* (Figure Identification-Psif and Digit Span Forward) (28,29), *executive function* (Digit Span Backward and Figure Logic) (28,29), *verbal fluency* (phonemic fluency derived from the Controlled Oral Word Association-FAS and semantic fluency derived from “animals” task) (30), and *visuospatial abilities* (Koh’s block test) (28). These domains were generated after standardizing raw scores from the single tests into *z*-scores (using mean and *SD* of the sample) and then averaging the *z*-scores across the tests within each domain. Finally, a composite score of global cognitive performance (*G*-score) was generated by averaging the *z*-scores of all 5 domains.

### Statistical Analysis

Sociodemographic, health, and cognitive characteristics between groups (no MetS vs MetS) were analyzed using chi-square test or *t*-test. Multivariable linear regressions with sandwich variance estimators were used to estimate the mean differences (β-coefficients) and 95% confidence intervals (95% CI) in cognitive performance among participants with MetS versus those without, using *G*-score as well as each domain as separate outcomes. Potential confounders included sex, education, physical activity, smoking, alcohol risk consumption, heart disease, stroke/TIA, and *APOE*-ε4 allele.

We investigated whether education, cardio- and cerebrovascular diseases (heart disease and stroke/TIA), and a major genetic risk factor for AD (the *APOE*-ε4 allele) could modify the association between MetS and cognitive function. Thus, interactions between MetS and each of the abovementioned factors were assessed by incorporating the 2 variables (MetS and the respective factor) and their cross-term product in the regression models. Stratified analyses by the factor (education, heart disease, stroke/TIA, or *APOE*-ε4) were also performed as well as joint exposure analyses of MetS and the possible protective/risk factor.

Given the role of atherosclerosis in the pathogenesis of metabolic diseases and cognitive aging alike (31), joint exposure analysis was further performed combining MetS (no vs yes) with claudication (no vs yes) into 4 groups: (i) those without MetS and claudication (“no disease”); (ii) those who had MetS but no claudication (“MetS only”); (iii) those who had claudication but no MetS (“Claudication only”); and (iv) those who had both diseases (“MetS and Claudication”).

A 2-sided *p* value of <.05 indicated statistical significance, except in the case of interactions analysis, where a *p* value of <.10 indicated the presence of a significant interaction (32). All statistical analyses were performed with Stata, version 16.0 (StataCorp LP, College Station, TX).

## Results

### Characteristics of the Study Population

Among the participants, 618 (54.6%) were identified with MetS. Participants’ sociodemographic and health characteristics by metabolic status are presented in Table 1. Overall, those with MetS were more likely to be men, have lower educational level, be less physically active, have heart disease and claudication, and worse cardiometabolic features than participants without MetS. On average, participants with MetS scored lower on all cognitive tests (Supplementary Table S1).

**Table 1.** Characteristics of Dementia-Free Participants (*n* = 1131) From the Gothenburg H70 Birth Cohort 1944 by the Presence/Absence of Metabolic Syndrome

Variable	Metabolic Syndrome		*p* *
	No (*n* = 513)	Yes (*n* = 618)	
Age, y	70.5 ± 0.26	70.6 ± 0.28	.069
Women	296 (57.7)	307 (49.7)	.007
Education, y	14.0 ± 4.08	12.8 ± 5.3	<.001
Primary school	49 (9.6)	105 (17.0)	<.001
Secondary school	222 (43.4)	321 (52.0)	
Higher education	242 (47.2)	191 (31.0)	
*APOE* status			
Any ɛ4 carriers	165 (32.9)	194 (32.6)	.911
One allele	140 (27.9)	180 (30.2)	.055
Two alleles	25 (5.0)	14 (2.4)	
MMSE score	29.2 ± 1.11	28.9 ± 1.26	<.001
Vascular risk factors			
Current/former smoking	306 (59.7)	398 (64.6)	.087
Alcohol risk consumption	149 (29.0)	196 (31.8)	.314
BMI, kg/m^2^	23.5 ± 3.13	28.2 ± 4.43	<.001
Underweight (<20)	61 (11.9)	1 (0.2)	<.001
Normal (≥20–25)	315 (61.4)	131 (21.3)	
Overweight (≥25–30)	120 (23.4)	309 (50.2)	
Obese (≥30)	17 (3.3)	174 (28.3)	
Physical inactivity	3 (0.6)	36 (6.0)	<.001
Medical conditions			
Heart disease	76 (14.8)	135 (21.8)	.003
Ischemic heart diseases	42 (8.2)	77 (12.5)	.020
Heart failure	3 (0.6)	21 (3.4)	.001
Atrial fibrillation	37 (7.2)	60 (9.7)	.136
Stroke/TIA	24 (6.7)	40 (9.8)	.122
Claudication	6 (1.2)	21 (3.4)	.015
Depression	43 (8.4)	56 (9.1)	.690
Cardiometabolic features			
Central adiposity	244 (47.6)	618 (100)	<.001
Lipid profile			
Raised triglycerides	72 (14.2)	360 (58.4)	<.001
Reduced HDL-cholesterol	16 (3.1)	124 (20.2)	<.001
Total cholesterol, mmol/L	5.74 ± 1.09	5.32 ± 1.20	<.001
LDL-cholesterol, mmol/L	3.59 ± 0.96	3.01 ± 1.06	<.001
Raised blood pressure	359 (70.0)	588 (95.2)	<.001
Hypertension	283 (55.2)	518 (83.8)	<.001
Raised plasma glucose	142 (28.0)	525 (85.5)	<.001
Diabetes	16 (3.2)	133 (21.7)	<.001
Cognitive domains			
*G*-score	0.11 ± 0.61	−0.11 ± 0.63	<.001
Episodic memory	0.08 ± 0.73	−0.08 ± 0.77	<.001
Attention/perceptual speed	0.12 ± 0.77	−0.09 ± 0.80	<.001
Executive function	0.10 ± 0.80	−0.08 ± 0.79	<.001
Verbal fluency	0.16 ± 0.87	−0.17 ± 0.87	<.001
Visuospatial abilities	0.09 ± 1.02	−0.07 ± 0.98	.008

*Notes: APOE*-ɛ4 = apolipoprotein E gene-ɛ4 allele; BMI = body mass index; HDL = high-density lipoprotein; LDL = low-density lipoprotein; MMSE = Mini-Mental State Examination; TIA = transient ischemic attack. Data are presented as means ± *SD* or number (proportion %). Missing data are as follows: education (*n* = 1), smoking (*n* = 2), alcohol risk consumption (*n* = 2), physical activity (*n* = 27), BMI (*n* = 3), stroke/TIA (*n* = 365), diabetes and raised plasma glucose (*n* = 9), depression (*n* = 4), *APOE*-ɛ4 (*n* = 33), raised triglycerides (*n* = 6), total cholesterol/HDL-c/LDL-c (*n* = 9), MMSE (*n* = 4), *G*-score (*n* = 2), episodic memory (*n* = 4), attention/perceptual speed (*n* = 15), executive function (*n* = 18), verbal fluency (*n* = 6), visuospatial abilities (*n* = 61).

*p < .05.

### MetS and Cognitive Functioning

Table 2 shows the mean differences in cognitive function of participants with MetS versus those without (reference). In basic-adjusted linear regressions (by sex and education), MetS was associated with poorer performance in global cognition (β −0.12 [95% CI −0.19, −0.06]) and in all cognitive domains except visuospatial abilities. After adjusting for smoking, alcohol risk consumption, cardio- and cerebrovascular diseases, and *APOE*-ε4, the associations remained similar, except for episodic memory that became no longer associated with MetS.

**Table 2.** Linear Regression β-Coefficients and 95% Confidence Intervals for the Association Between Metabolic Syndrome and Composite Cognitive Domain

Composite Domains	Model 1*		Model 2†		Model 3‡	
	β (95% CI)	*p*	β (95% CI)	*p*	β (95% CI)	*p*
*G*-score	−0.12 (−0.19, −0.06)	<.001	−0.12 (−0.20, −0.04)	.003	−0.13 (−0.21, −0.04)	.003
Episodic memory	−0.10 (−0.18, −0.01)	.027	−0.08 (−0.19, 0.02)	.114	−0.08 (−0.18, 0.03)	.149
Attention/perceptual speed	−0.14 (−0.23, −0.05)	.003	−0.13 (−0.24, −0.02)	.024	−0.14 (−0.25, −0.02)	.016
Executive function	−0.09 (−0.19, −0.004)	.041	−0.12 (−0.23, −0.004)	.041	−0.12 (−0.23, −0.01)	.037
Verbal fluency	−0.21 (−0.30, −0.11)	<.001	−0.18 (−0.29, −0.06)	.002	−0.19 (−0.30, −0.08)	.002
Visuospatial abilities	−0.07 (−0.19, 0.04)	.223	−0.11 (−0.24, 0.03)	.130	−0.10 (−0.24, 0.04)	.142

*Notes: APOE*-ɛ4 = apolipoprotein E gene-ɛ4 allele; CI = confidence intervals; *G*-score = composite score for global cognition; TIA = transient ischemic attack. In all models, the reference group included participants without metabolic syndrome.

*Model 1: adjusted for sex and education.

^†^Model 2: adjusted for sex, education, physical activity, smoking, alcohol risk consumption, heart disease, and stroke/TIA.

^‡^Model 3: adjusted for sex, education, physical activity, smoking, alcohol risk consumption, heart disease, stroke/TIA, and *APOE*-ɛ4.

### The Role of Education and *APOE*-ε4 in the MetS–Cognition Association

We examined whether education and *APOE*-ε4 allele status modified the relationship between MetS and cognitive function. Statistically significant interactions were observed between MetS and education in the association with verbal fluency (*p* = .052), as well as between MetS and *APOE*-ε4 in the association with attention/perceptual speed (*p* = .030).

Stratified analyses by education revealed that among participants with higher education, those with MetS had poorer performance on global cognition (β −0.19 [95% CI −0.32, −0.05]), attention/perceptual speed (β −0.25 [95% CI −0.43, −0.08]), and verbal fluency (β −0.26 [95% CI −0.46, −0.07]) than those without MetS (Table 3). Such associations were not observed in people with lower educational attainment, nor in relation to episodic memory performance and visuospatial abilities. Further analyses within the participants with MetS showed that higher education, compared with primary education, was associated with better performance on global cognition (β 0.50 [95% CI 0.30; 0.70], *p* < .001), attention/perceptual speed (β 0.42 [95% CI 0.15; 0.69], *p* = .002), executive function (β 0.50 [95% CI 0.22; 0.77], *p* < .001), and verbal fluency (β 0.48 [95% CI 0.22; 0.74], *p* < .001), suggesting a possible protective role of high education in the MetS-related cognitive dysfunction.

**Table 3.** Associations Between Metabolic Syndrome and Cognitive Function From Stratified Analyses by Education (Primary, Secondary, High), Heart Disease (No, Yes), or Apolipoprotein E-ε4 Status (Noncarriers, Carriers)

	*n*	*G*-score		Attention/Perceptual Speed		Executive Function		Verbal Fluency	
		β (95% CI)	*p*	β (95% CI)	*p*	β (95% CI)	*p*	β (95% CI)	*p*
Primary school									
No MetS	49	Reference		Reference		Reference		Reference	
MetS	105	0.05 (−0.18; 0.28)	.653	0.04 (−0.31; 0.39)	.817	−0.01 (−0.34; 0.32)	.968	0.19 (−0.19; 0.56)	.330
Secondary school									
No MetS	222	Reference		Reference		Reference		Reference	
MetS	321	−0.12 (−0.24; 0.29)	.056	−0.08 (−0.25; 0.08)	.311	−0.14 (−0.30; 0.02)	.078	−0.20 (−0.36; −0.03)	.020
Higher education									
No MetS	242	Reference		Reference		Reference		Reference	
MetS	191	−0.19 (−0.32; −0.05)	.007	−0.25 (−0.43; −0.08)	.005	−0.14 (−0.33; 0.05)	.150	−0.27 (−0.46; −0.07)	.009
*APOE*-ε4 noncarriers									
No MetS	337	Reference		Reference		Reference		Reference	
MetS	402	−0.16 (−0.26; −0.06)	.002	−0.24 (−0.37; −0.11)	<.001	−0.15 (−0.28; −0.01)	.032	−0.19 (−0.33; −0.04)	.010
*APOE*-ε4 carriers									
No MetS	165	Reference		Reference		Reference		Reference	
MetS	194	−0.06 (−0.21; 0.09)	.450	0.10 (−0.12; 0.32)	.374	−0.06 (−0.26; 0.14)	.562	−0.20 (−0.42; 0.02)	.073

*Notes: APOE*-ε4, apolipoprotein E gene-ε4 allele; CI = confidence intervals; *G*-score, composite score for global cognition; MetS = metabolic syndrome. Linear regression models (separate for each cognitive outcome) were adjusted for sex, education, physical activity, smoking, alcohol risk consumption, heart disease, stroke/transient ischemic attack, and *APOE*-ε4.

In stratified analyses by *APOE*-ε4 status, the independent negative relationships of MetS with global cognition and the domains of attention/perceptual speed, executive function, and verbal fluency were present in participants who did not carry an ɛ4 allele (Table 3).

### Role of Comorbid Heart Disease and Claudication in the MetS–Cognition Association

**Figure 1.**
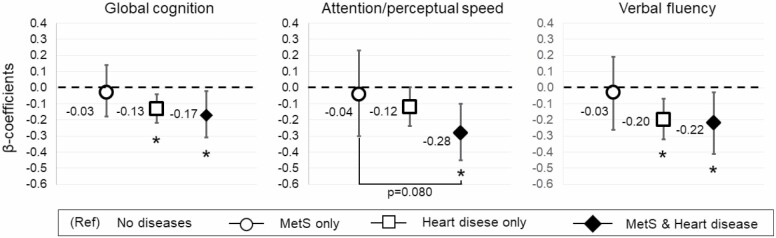
Joint association of MetS and heart disease in relation to cognitive function. The figure shows the multiadjusted (by sex, education, physical activity, smoking, alcohol risk consumption, stroke/TIA, and *APOE*-ε4) β-coefficients estimated from 3 separate linear regression models for the association between MetS and heart disease in relation to global cognition, attention/perceptual speed, and verbal fluency. “MetS only” indicates the group who had MetS but no heart disease (*n* = 483), “Heart disease only” indicates the group who had heart disease but no MetS (*n* = 76), and “MetS and Heart disease” indicates the group with both diseases (*n* = 135). The reference group was “No disease” including people without MetS and without heart disease (*n* = 437). *p value < .05 (reference group = no diseases). *APOE*-ε4 = apolipoprotein E gene-ε4 allele; MetS = metabolic syndrome.

Stratified analysis by heart disease showed poorer cognitive performance associated with MetS only in people without heart disease (Supplementary Table S2). Given that heart disease and individual components of MetS (eg, diabetes, obesity, hypertension) often coexist in the older population, we examined whether comorbid MetS and heart diseases exacerbate cognitive dysfunction. In joint analyses, comorbid MetS and heart diseases appeared to worsen cognitive performance in global cognition (β −0.17 [95% CI −0.31, −0.02]) and attention/perceptual speed (β −0.28 [95% CI −0.45, −0.10]) (Figure 1; Supplementary Table S3). Furthermore, the difference in attention/perceptual speed performance between the group with MetS and heart disease versus only MetS was nearly statistically significant (β −0.16 [95% CI −0.33, 0.01]; *p* = .080), suggesting that the combined impact of MetS and heart disease on attention/perceptual speed could be worse than having only MetS.

**Figure 2.**
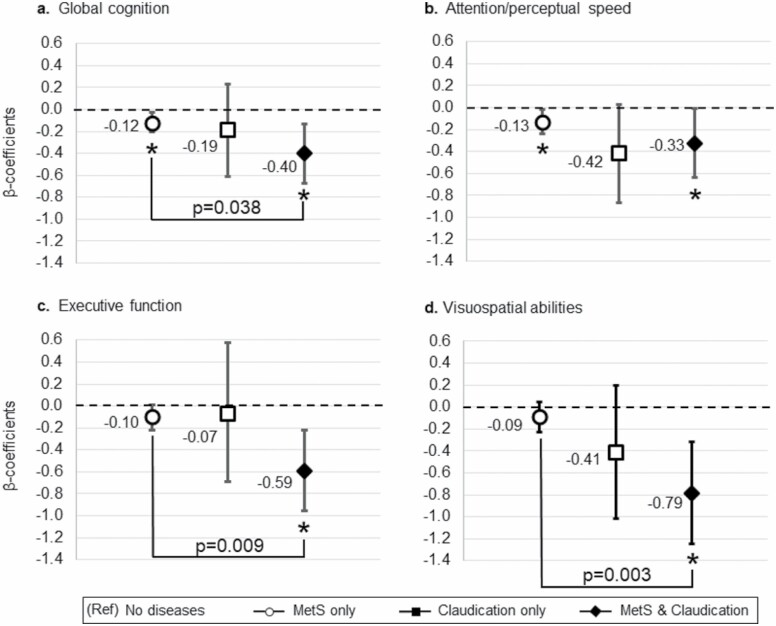
Joint association of MetS and claudication in relation to cognitive function. The figure shows the multiadjusted (by sex, education, physical activity, smoking, alcohol risk consumption, heart disease, stroke/TIA, and *APOE*-ε4) β-coefficients estimated from 4 separate linear regression models for the association between MetS and claudication in relation to global cognition (A), attention/perceptual speed (B), executive function (C), and visuospatial abilities (D). “MetS only” indicates the group who had MetS but no claudication (597), “Claudication only” indicates the group who had claudication but no MetS (*n* = 6), and “MetS and Claudication” indicates the group with both diseases (*n* = 21). The reference group was “No disease” including people without MetS and without claudication (*n* = 507). *p value < 0.05 (reference group = no diseases). *APOE*-ε4 = apolipoprotein E gene-ε4 allele; MetS = metabolic syndrome; TIA = transient ischemic attack.

Finally, we assessed the joint association of MetS and claudication in relation to cognition. Figure 2 shows the mean differences in cognitive performance according to the combination of MetS and claudication (reference group: “no disease”). MetS combined with claudication was associated with poorer global cognition (β −0.40 [95% CI −0.20, −0.03]), attention/perceptual speed (β −0.33 [95% CI −0.24, −0.02]), executive function (β −0.59 [95% CI −0.96, −0.22), and visuospatial (β −0.79 [95% CI −1.25, −0.32) performance than being without MetS and not having claudication or having only MetS.

For participants with MetS, comorbid claudication was associated with poor performance in global cognition (β −0.30 [95% CI −0.59, −0.02]), executive function (β −0.50 [95% CI −0.86, −0.11]), and visuospatial abilities (β −0.71 [95% CI −1.18, −0.24]).

### Supplementary Analyses

We tested whether cerebrovascular disorders or physical inactivity modified the associations between MetS and domain-specific cognitive function. We found no interaction or differences in stratified analyses by stroke/TIA or physical inactivity in relation to cognition.

Separate linear regression models to examine the joint associations between MetS and education, as well as between MetS and *APOE*-ε4 were also performed (Supplementary Table S4). Primary education (lowest educational attainment) was associated with the lowest cognitive performance (regardless of MetS status). However, the group with higher education and MetS showed poorer cognitive performance than the group with high education and no MetS (β −0.20 [95% CI −0.33; −0.07]). Having MetS and carrying any ε4 allele was associated with low cognitive performance in all domains except episodic memory and visuospatial abilities.

To address cognitive function at the early stages of a possible cognitive deterioration, we further excluded participants with an MMSE score < 27 (*n* = 55); the associations between MetS and domain-specific cognitive performance remained unchanged.

We compared the sociodemographic and health-related characteristics between participants (*n* = 1131) versus those who were excluded (*n* = 72). The latter group was more likely to have lower education (50.0% vs 13.6% among participants), be less physically active (34.8% vs 3.5% among participants), have a history of stroke/TIA (26.7% vs 8.4% among participants), and claudication (7.7% vs 2.4% among participants). No differences were observed in relation to MetS, heart disease, or *APOE*-ε4 allele.

In sensitivity analysis (see Supplementary Appendix B), we repeated all the analyses using an alternative definition of MetS (by the *International Diabetes Federation Task Force on Epidemiology and Prevention*; *National Heart, Lung, and Blood Institute*; *American Heart Association*; *World Heart Federation*; *International Atherosclerosis Society*; and *International Association for the Study of Obesity*) that emphasizes the role of insulin resistance as a major underlying mechanism. Results were unchanged.

Given the evidence of a link between body mass index and cognition, we examined the association of body mass index (normal [≥20–25 kg/m^2^] vs overweight/obese [≥25 kg/m^2^]) with cognitive outcomes, regardless of the metabolic status. In addition, associations of the individual MetS components (raised triglycerides, reduced high-density lipoprotein cholesterol, raised blood pressure, and raised blood glucose) with cognitive outcomes were analyzed. Results showed that overweight/obesity, raised triglycerides, and reduced high-density lipoprotein cholesterol were associated with poor global and domain-specific (attention/processing speed and executive function) performance. Although no statistically significant associations emerged in relation to raised blood pressure and raised blood glucose, the overt chronic disease of diabetes and isolated systolic hypertension (systolic blood pressure ≥ 130 mm Hg)—which are part of the MetS together with their preclinical stages of prediabetes and prehypertension—were associated with poorer global cognition, attention/perceptual speed, and/or executive function. Besides supporting the relevance of clustered cardiometabolic disease in relation to cognitive dysfunction and dementia, these findings indicate that future studies of MetS in older population should consider overt cardiometabolic disease.

Finally, multiple imputation by chained equations for missing values on the covariates was performed; results were unchanged.

## Discussion

In this population-based cohort of septuagenarians without dementia, MetS was associated with poorer cognitive performance, particularly in the domains of attention/perceptual speed, executive function, and verbal fluency (fluid abilities). Fluid abilities rely on the frontal-lobe function, which is vulnerable to vascular brain changes, especially during aging (33,34). These associations were only detected in people without *APOE*-ε4 allele or highly educated. However, among those who had MetS, a high level of education was related to better cognitive performance. This suggests that high education may help preserve cognitive health, even when major risk factors for dementia cluster together (eg, in MetS). Finally, comorbid heart diseases or claudication seemed to exacerbate the harmful impact of MetS on cognitive function.

Over the last decade, a number of studies have investigated the association between MetS and cognitive function (7,8,35). Although the majority observed an association between MetS and global cognitive deficits (7,8,36), there is less consensus on the specific domains that are impaired. Recently, a large systematic review reported a consistent association between MetS and deficits in executive function without a predominant verbal component, whereas findings on the other core cognitive domains (verbal fluency, episodic memory, perceptual speed, or visuospatial abilities) were less consistent (7). In line with previous findings, we detected an association between MetS and executive function but also with other frontal-lobe functions such as attention/perceptual speed and verbal fluency. Moreover, our study confirmed a lack of association between MetS and episodic memory, which is a hallmark of typical AD neurodegeneration (34). Such neuropsychological profile is typical of vascular cognitive impairment, especially of subcortical origin. Vascular cognitive impairment refers to the entire spectrum of cognitive disorders—from milder forms of cognitive dysfunction to fully-developed dementia—due to vascular neuropathology (both large and small vessel disease) (14,33). Our findings suggest that MetS may increase the risk of preclinical vascular cognitive impairment. This hypothesis, however, needs to be tested in cohorts with available neuropsychological testing and neuroimaging markers of vascular brain pathology. Discrepancies between our findings and those from prior studies could in part reflect methodological factors, such as the variability in the criteria used and implemented to identify MetS; inclusion of participants in the prodromal stage of dementia; different age ranges of the study participants; or use of single tests instead of composite measures to assess cognitive domains, with consequent increases of measurement and type I errors (37). Future longitudinal studies need to investigate how these decrements in cognition evolve over time and disentangling whether they are part of the normal cognitive aging or whether they represent a preclinical manifestation of dementia.

To the best of our knowledge, this is one of the first studies to highlight that cardiovascular comorbidities could be an important coplayer in the relationship between MetS and cognition. Indeed, we showed that comorbid MetS and cardiovascular disease (ie, heart disease or claudication) were associated with worsened cognitive performance than either disease alone. This is clinically relevant because older adults with MetS have twice the risk of developing cardiovascular disease than those without MetS (38), and the presence of cardiovascular disease (eg, atrial fibrillation, ischemic heart disease, heart failure) increases the risk of dementia (39). On the other hand, both MetS and cardiovascular disease are potentially modifiable themselves, through behavioral changes to healthy lifestyles and/or pharmacological treatment. As such, these 2 conditions represent promising targets for interventions to reverse or slow down the clinical manifestation of dementia. Moreover, our findings on a possible exacerbating role of claudication give us more insight into the biological mechanisms underlying MetS-related cognitive dysfunction, advocating for a substantial role of atherosclerotic changes. Biologically, multiple pathways could underlie the association between MetS and cognitive dysfunction (35,40). These pathways may stem primarily from dyslipidemia and hyperglycemia, coupled with insulin resistance (ie, a hallmark of MetS), and act on biochemical and systemic levels in the body. Through a cascade of events involving lipidemic dysregulation, oxidative stress, and inflammation, MetS could gradually lead to dysfunction of the blood–brain barrier, chronic hypoperfusion, damage of the large and small cerebral vessels and, over time, to cognitive impairment and dementia (33,40). Future research needs to better understand to what extent MetS and cardiovascular comorbidities synergize in the chain of events leading to a rapid cognitive deterioration and dementia.

Our results also showed the negative impact of having MetS on fluid abilities only among *APOE*-ε4 noncarriers. Similar findings have been described in 2 Swedish cohorts addressing the link between pre/diabetes (1 of the 4 components of MetS) and cognitive performance (41,42), but not in relation to MetS. The *APOE*-ε4 allele is a major genetic risk factor for late-onset AD (41). A possible interpretation is that *APOE*-ε4 carriers may have already accumulated sufficient AD pathology to manifest dementia symptoms earlier than noncarriers. Among noncarriers, other physiological insults (eg, the vascular and metabolic dysregulations inherent in MetS) could be needed to manifest cognitive dysfunction and eventually progress to more severe forms of vascular cognitive impairment. Taken together, a cognitive profile characterized by deficits of the frontal-lobe functions and relatively preserved episodic memory, only among *APOE*-ε4 noncarriers, as well as the exacerbating role of cardiovascular comorbidities (heart disease and claudication), point toward the existence of vascular brain pathology as a potential driver of cognitive dysfunction related to MetS—at least in the initial stage of cognitive deterioration. Whether the vascular pathology is the sole driver or interacts with other neuropathology is unknown. Indeed, dementia is the end stage of several years or decades during which multiple biological processes interact to trigger and accumulate diverse neuropathological changes (33,43). Accumulating evidence from autopsy and in vivo studies are highlighting more and more that neurodegenerative (eg, β-amyloid, tau, α-synuclein, and TDP-43) and vascular brain pathology often coexist in the aging brain (33). Unfortunately, cognitive profiles that rely only on neuropsychological testing do not take brain pathologies into account. In the future, more research integrating in vivo neuroimaging with postmortem neuropathological data as well as cerebrospinal fluid, cognitive, and clinical information is warranted to better understand the interplay between neurodegenerative and vascular brain pathology in the MetS–cognition link.

Finally, we detected negative associations between MetS and cognitive performance in highly educated participants, but not in those with lower educational attainment. It is plausible that older people with low formal education may have already clinically expressed dementia, whereas those with high education remained in the preclinical phase for a longer time. This hypothesis is partly supported by the high proportion of people with low education who were excluded because of dementia in our sample. Whereas low education is an established risk factor for dementia, high formal education has been linked to cognitive benefits and reduced risk of dementia (4,44). Education represents a major source of cognitive stimulation during childhood and adolescence (44). As such, it may supply resilience against dementia helping the individual to cope with brain pathologies, thereby preserving cognitive function (45). However, with advancing age, protective (eg, education) and risk factors (eg, MetS) for dementia interact, and the end stage of this interplay is unknown. Based on our results, it can be speculated that older people with high formal education may require additional physiological insults, such as an accumulation of vascular pathology (possibly underlying MetS), to wash off the compensatory benefits of education and express cognitive deficits. On the other hand, our study shows that among older people with MetS, high formal education was related to better cognitive performance. This finding adds a piece to the puzzle about the existence of “residual” compensatory mechanisms in individuals at risk of developing dementia (46,47), in particular among those with coexisting cardiovascular and metabolic disorders. Longitudinal research is needed to better understand the extent to which other lifetime sources of stimulation (eg, occupation, leisure activities, and social network), individually and/or aggregated, can promote cognitive health in the presence of age-related cardiometabolic disorders.

Novelty and strength of this study are the investigation of the interplay between co-occurring risk factors (ie, as part of the MetS but also with age-related cardiovascular comorbidities) for dementia and early-life compensatory factors in relation to cognition—possibly tackling the preclinical phase of cognitive deterioration—that has not been done before. Furthermore, the results are from a well-characterized population-based sample, which was systematically selected based on birthdates. This aspect together with the novelty and the comprehensive medical and cognitive assessment carried out by health professionals and trained staff are the strengths of the current study. Specifically, the clinical diagnosis of dementia and the detailed cognitive assessment addressing 5 core cognitive domains allowed us to focus on cognitive decrements in a preclinical phase of dementia. The use of composite scores to measure cognitive domains rather than relying on single tests has the advantage of limiting type I error and increasing reliability of the findings (37). Some limitations need to be acknowledged. The cross-sectional design prevented the establishment of a temporal relationship between MetS and cognitive function. Participants were generally healthier and higher educated than those excluded (sensitivity analysis); thus, selectivity toward healthy septuagenarians, when compared with the general population, could have led to an underestimation of the observed associations. The focus of this study was on the preclinical stage of dementia, when the pathological damage is present but has not yet extensively accumulated, thus representing an ideal time window for preventive strategies. To best fulfill our purpose, in the absence of biomarkers, we further excluded participants with an MMSE score < 27 in the sensitivity analyses and results were unchanged. As in any observational study, residual confounding cannot be completely ruled out; however, our results seemed robust to the adjustment for multiple confounding factors as well as after sensitivity analysis. Moreover, the identification of MetS integrating different data sources (self- and proxy-reports, medical records from the NPR, and laboratory tests) increased the likelihood to detect the presence of diseases, thus reducing the possibility of differential misclassification of the exposure. Finally, the results of this study can be generalized to populations with similar characteristics as the H70 cohort, although the mechanistic insight may be generalized to the aging population with broader age ranges.

In summary, our findings highlight that coexisting, possibly modifiable or controllable, metabolic and cardiovascular conditions could impair cognition, likely starting with frontal-lobe dependent functions such as fluid abilities that are susceptible to vascular brain insults. However, early-life resilience-enhancing factors may mitigate the harmful impact of MetS, thereby preserving cognition. Our study points toward a predominant role of vascular neuropathology underlying the effects of MetS on cognition, but future studies need to investigate in-depth the biological mechanisms behind these associations posing a particular focus on the interplay between multiple brain pathologies and compensatory mechanisms. This will aid earlier and more precise diagnoses of cognitive impairment and provide key insights for the development of more personalized interventions to prevent dementia in at-risk populations.

## Supplementary Material

glab195_suppl_Supplementary_Materials
