# Supplementary material for: Metabolic Syndrome Is Associated With Poor Cognition: A Population-Based Study of 70-Year-Old Adults Without Dementia
Source: J Gerontol A Biol Sci Med Sci. 2021 Jul 6;76(12):2275–83. doi: 10.1093/gerona/glab195 (PMC8599084; doi:10.1093/gerona/glab195)
Supplement: glab195_suppl_Supplementary_Materials [file glab195_suppl_supplementary_materials.pdf]

ONLINE-ONLY SUPPLEMENTAL MATERIAL

1) APPENDIX A. Assessment of cardio- and cerebro-vascular diseases and claudication.
2) APPENDIX B. Sensitivity analysis using the definion of MetS proposed by the *International Diabetes Federation Task Force on Epidemiology and Prevention; National Heart, Lung, and Blood Institute; American Heart Association; World Heart Federation; International Atherosclerosis Society; and International Association for the Study of Obesity*.
3) eFigure 1. Flowchart of the baseline (2014-2016) study population in the Gothenburg H70 Birth Cohort Studies–Birth cohort 1944.
4) eTable 1. Cognitive characteristics of dementia-free participants (n=1131) by presence/absence of metabolic syndrome.
5) eTable 2. Associations between metabolic syndrome (Mets) and cognitive function from stratified analyses by heart disease.
6) eTable 3. Joint association between metabolic syndrome (MetS) and education, MetS and heart diseases, and MetS and apolipoprotein status in relation to cognitive function.
7) eTable 4. Joint association between metabolic syndrome (MetS) and education, MetS and heart diseases, and MetS and apolipoprotein status in relation to cognitive function.

APPENDIX A. Assessment of cardio- and cerebro-vascular diseases and claudication.

CVD included myocardial infarction, angina pectoris, heart failure, atrial fibrillation, and stroke/transient ischemic attack (TIA). Specifically, a standard 12-lead electrocardiogram (ECG) was coded, according to the Minnesota Code, by a biomedical analyst working at the cardiac laboratory at Sahlgrenska University Hospital (Rydberg Sterner et al. 2019). Myocardial infarction was identified based on self-reported history or the presence of moderate or major Q-waves on ECG (Minnesota Codes: 1-1-X or 1-2-X, excluding 1-2-6 and 1-2-8). Angina pectoris was based on self-reported history or the standard criteria (*Rose GA. The diagnosis of ischaemic heart pain and intermittent claudication in field surveys. Bull World Health Organ 1962;27:645-658*). Heart failure was based on self-reported history. Atrial fibrillation was based on self-reported history or ECG (Minnesota Code: 8-3). Stroke and TIA—defined as sudden onset of focal symptoms or aphasia lasting for more than 24 hours (stroke) or less than 24 hours (TIA)—were identified based on self-report, key informants interview, or the Swedish National Patient register (ICD-10 codes: I60, I61, I629, I630–I635, I638–I639, I64, I690–I691, and I693–I694).

The presence of intermittent claudication was assessed based on the criteria proposed by Rose in 1962 (*Rose GA. The diagnosis of ischaemic heart pain and intermittent claudication in field surveys. Bull World Health Organ 1962;27:645-658*) with two questions (no vs. yes): 1) Do you usually get pain in the calves when you walk uphill, in stairs or on the level?; 2) Does the pain disappear within a few minutes if you stop to rest without sitting down?. Participants who answered "yes" to both questions were categorized as having claudication.

APPENDIX B. Sensitivity analysis.

In 2009, the *International Diabetes Federation Task Force on Epidemiology and Prevention; National Heart, Lung, and Blood Institute; American Heart Association; World Heart Federation; International Atherosclerosis Society; and International Association for the Study of Obesity* (Alberti KG, Eckel RH, Grundy SM, et al. Circulation 2009, 120(16):1640-5; doi 10.1161/CIRCULATIONAHA.109.192644) proposed an alternative definition of MetS that emphasize the role of insulin-resistance as a major underlying mechanisms. Thus, according to the abovementioned criteria, MetS was defined as the presence of at least three of the following five factors: central adiposity, raised triglycerides, reduced high-density lipoprotein cholesterol, raised blood pressure, and raised blood glucose. We repeated all the analyses in the manuscript using this definition. Results were substantially unchanged.

Briefly, the 645 (57%) participants identified with the insulin-resistance-centred definition of MetS were more likely to be men, have lower educational level, be less physically active, have heart disease and claudication, worse cardiometabolic features, and lower performance on all cognitive domains than participants without MetS. Multi-adjusted linear regression models (by sex, education, smoking, alcohol risk consumption, cardio- and cerebro-vascular diseases, and *APOE*-ε4) showed that MetS was independently associated with poorer performance in global cognition (β -0.13 [95% CI -0.21, -0.05], p=0.003), attention/perceptual speed (β -0.16 [95% CI -0.27; -0.05], p=0.005), executive function (β -0.12 [95% CI -0.24; -0.01], p=0.033), and verbal fluency (β -0.19 [95% CI -0.31; -0.73], p=0.002).

Stratified analyses by education replicated the negative associations of MetS with global cognition, attention/perceptual speed, and verbal fluency among participants with higher education, but not with a lower educational attainment. Similarly, stratified analyses by *APOE*-ε4 (carriers vs. non-carriers of any ε4 allele) showed that the independent negative relationships of MetS with global cognition and the domains of attention/perceptual speed, executive function, and verbal fluency were present in participants who did not carry an ε4 allele.

Finally, joint analyses showed that participants with comorbid MetS and heart diseases had worse cognitive performance in global cognition (β -0.17 [95% CI -0.31, -0.03], p=0.015), attention/perceptual speed (β -0.32 [95% CI -0.49; -0.15], p<0.001), executive function (β -0.18 [95% CI -0.36; -0.001], p=0.049), and verbal fluency (β -0.20 [95% CI -0.39; -0.01], p=0.034). Also, participants with comorbid MetS and claudication had poorer performance in global cognition (β -0.411 [95% CI -0.68, -0.14], p=0.003), attention/perceptual speed (β -0.35 [95% CI -0.66; -0.03], p=0.032), executive function (β -0.60 [95% CI -0.97; -0.23], p=0.002), and visuospatial abilities (β -0.79 [95% CI -1.25; -0.32], p=0.001).

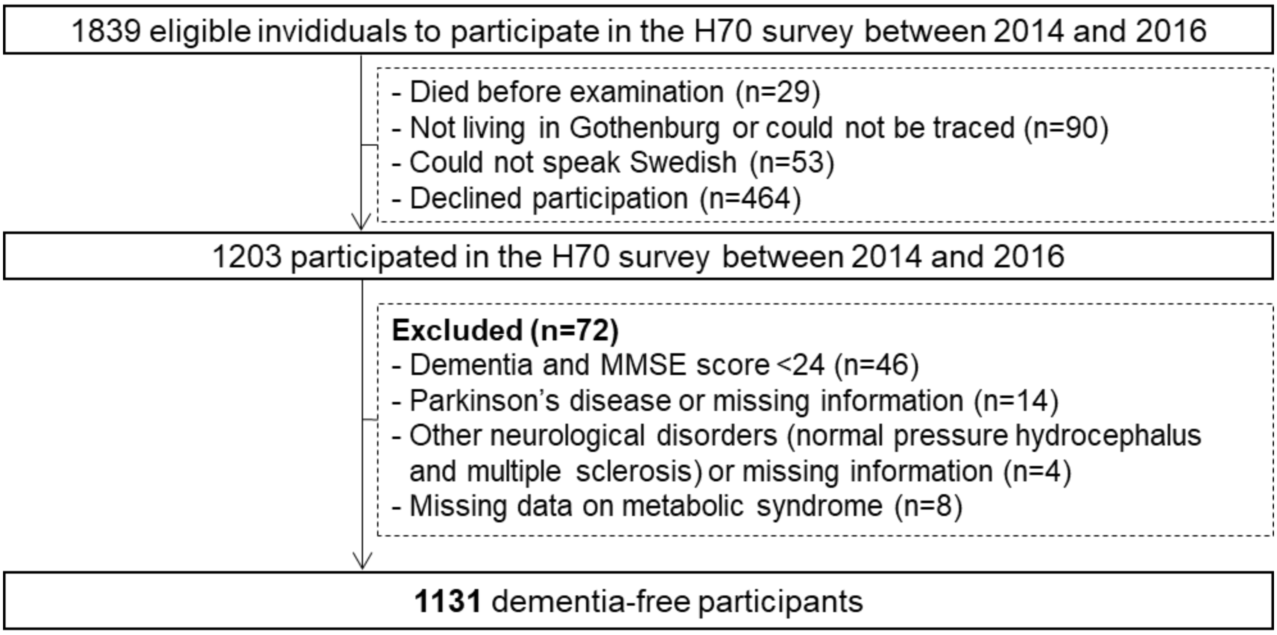

eFigure 1. Flowchart of the baseline (2014-2016) study population in the Gothenburg H70 Birth Cohort Studies–Birth cohort 1944.

Abbreviations: MMSE. Mini-Mental State Examination

eTable 1. Cognitive characteristics of dementia-free participants (n=1131) by presence/absence of metabolic syndrome.

Cognitive tests	n (min–max)	Metabolic syndrome		*p* value
		No (n=513)	Yes (n=618)	
Episodic memory				
Memory-in-Reality, free recall	1099 (0–10)	8.73 ± 1.44	8.45 ± 1.61	0.002
Thurstone's Picture Memory	1028 (2–28)	22.2 ± 4.09	21.8 ± 4.10	0.059
12-object, delayed recall	1121 (0–12)	7.61 ± 1.86	7.27 ± 1.86	0.003
Attention/perceptual speed				
Figure Identification–Psif	1107 (3.5–57.5)	28.1 ± 7.96	26.7 ± 8.03	0.003
Digit Span Forward	1068 (1–8)	6.08 ± 1.17	5.81 ± 1.16	<0.001
Executive function				
Digit Span Backward	1105 (0–8)	4.55 ± 1.21	4.27 ± 1.24	<0.001
Figure Logic	1093 (0–30)	20.1 ± 4.41	19.6 ± 4.28	0.062
Verbal fluency				
Semantic fluency	1122 (6–49)	24.9 ± 6.27	23.1 ± 6.36	<0.001
Phonemic fluency	1054 (4–110)	43.6 ± 14.3	38.5 ± 14.5	<0.001
Visuospatial abilities				
Koh's Block test	1070 (0–41)	21.2 ± 7.09	20.0 ± 6.86	0.008

Note: Data are presented as means ± standard of the raw test scores.
Abbreviations: *APOE*-ε4. apolipoprotein ε4 allele; HDL. High-density lipoprotein; LDL. Low-density lipoprotein.
Missing data: Memory-in-Reality=32, Thurstone's Picture Memory=103, 12-object' word list delay recall=10, Figure Identification=24, Digit Span Forward=63, Digit Span Backward=26, Figure Logic=38, Semantic fluency=9, Phonemic fluency=77, Koh's Block test=61.

eTable 2. Associations between metabolic syndrome (Mets) and cognitive function from stratified analyses by heart disease.

		G-score		Attention/ perceptual speed		Executive function		Verbal fluency	
	**n**	**β (95% CI)**	**p**	**β (95% CI)**	**p**	**β (95% CI)**	**p**	**β (95% CI)**	**p**
No heart diseases									
No MetS	437	Reference		Reference		Reference		Reference	
MetS	483	-0,13 (-0,22; -0,04)	0,005	-0,12 (-0,24; -0,001)	0,050	-0,13 (-0,25; -0,01)	0,047	-0,19 (-0,32; -0,06)	0,004
Heart disease									
No MetS	76	Reference		Reference		Reference		Reference	
MetS	135	-0,13 (-0,33; 0,06)	0,178	-0,19 (-0,50; 0,11)	0,213	-0,13 (-0,41; 0,16)	0,386	-0,19 (-0,48; 0,08)	0,160

Linear regression models, separate for each cognitive outcome, were adjusted for sex, education, physical activity, smoking, alcohol risk consumption, heart disease, stroke/TIA, and *APOE*-ε4.
Abbreviations: *APOE*-ε4, apolipoprotein E gene-ε4 allele; CI, confidence intervals; TIA, transient ischemic attack.

eTable 3. Joint association between metabolic syndrome (MetS) and education, MetS and heart diseases, and MetS and apolipoprotein status in relation to cognitive function.

			G-score	Attention/ perceptual speed	Executive function	Verbal fluency
**Heart disease**	**MetS**	**n**	**β (95% CI)**	**β (95% CI)**	**β (95% CI)**	**β (95% CI)**
No	No	437	Reference	Reference	Reference	Reference
Yes	No	76	-0,03 (-0,18; 0,14)	-0,04 (-0,30; 0,23)	-0,04 (-0,27; 0,18)	-0,03 (-0,26; 0,19)
No	Yes	483	-0,13 (-0,22; -0,04) *	-0,12 (-0,24; 0,001)	-0,13 (-0,25; -0,004) †	-0,20 (-0,32; -0,07) †
Yes	Yes	135	-0,17 (-0,31; -0,02) †	-0,28 (-0,45; -0,10) †	-0,17 (-0,36; 0,02)	-0,22 (-0,41; -0,03) †

β-coefficients and 95% Confidence Intervals (CI) from three separate linear regression models adjusted for sex, education, physical activity, alcohol risk consumption, stroke, and *APOE*-ε4,
* $p \leq 0,001$; † $p < 0,05$

eTable 4. Joint association between metabolic syndrome (MetS) and education, MetS and heart diseases, and MetS and apolipoprotein status in relation to cognitive function.

			G-score	Attention/ perceptual speed	Executive function	Verbal fluency
**Factor**	**MetS**	**n**	**β (95% CI)**	**β (95% CI)**	**β (95% CI)**	**β (95% CI)**
Education						
High	No	242	Reference	Reference	Reference	Reference
Secondary	No	222	-0,41 (-0,53; -0,29) *	-0,42 (-0,58; -0,27) *	-0,45 (-0,62; -0,28) *	-0,50 (-0,64; -0,28) *
Primary	No	49	-0,73 (-0,92; -0,55) *	-0,68 (-0,94; -0,41) *	-0,68 (-0,91; -0,45) *	-0,88 (-1,19; -0,56) *
High	Yes	191	-0,20 (-0,33; -0,07) †	-0,24 (-0,41; -0,07) †	-0,16 (-0,35; 0,03)	-0,29 (-0,47; -0,10) †
Secondary	Yes	321	-0,52 (-0,64; -0,41) *	-0,51 (-0,66; -0,35) *	-0,57 (-0,73; -0,42) *	-0,64 (-0,81; -0,48) *
Primary	Yes	105	-0,72 (-0,91; -0,53) *	-0,67 (-0,92; -0,42 *	-0,69 (-0,95; -0,43) *	-0,77 (-1,01; -0,53) *
APOE-ε4						
Non-carriers	No	337	Reference	Reference	Reference	Reference
Carriers	No	165	-0,02 (-0,14; 0,11)	-0,12 (-0,29; 0,04)	-0,06 (-0,23; 0,11)	-0,03 (-0,21; 0,14)
Non-carriers	Yes	402	-0,15 (-0,25; -0,05) †	-0,23 (-0,35; -0,10) *	-0,15 (-0,28; -0,01) ǁ	-0,18 (-0,32; -0,04) †
Carrier	Yes	194	-0,12 (-0,24; 0,01)	-0,08 (-0,26; 0,09)	-0,15 (-0,31; 0,02)	-0,26 (-0,43; -0,09) †

β-coefficients and 95% Confidence Intervals (CI) from three separate linear regression models adjusted for sex, physical activity, alcohol risk consumption, stroke, and the remaining covariates (education, heart disease, or *APOE*-ε4),
* $p \leq 0,001$; † $p \leq 0,01$; ǁ $p < 0,05$.
